# Prevalence of erectile dysfunction among healthy and sexually active Malaysian men aged 18 to 39 years

**DOI:** 10.1093/sexmed/qfag009

**Published:** 2026-04-14

**Authors:** Arvinder-Singh HS, Rukman Awang Hamat, Josephine P Durai, Harpreet Kaur, Nuh I Razlan, Sivaneswaran Lechmiannandan, Christopher C K Ho, Philip R Devesahayam

**Affiliations:** Clinical Research Centre, Institute of Clinical Research (NIH), Hospital Raja Permaisuri Bainun Ipoh, 30450, Perak, Ministry of Health, Malaysia; Faculty Research Support Unit and Deputy Dean's Office (International and Research), Faculty of Medicine and Health Sciences, Universiti Putra Malaysia (UPM), Serdang, Selangor, Ministry of Higher Education, Malaysia; Faculty Research Support Unit and Deputy Dean's Office (International and Research), Faculty of Medicine and Health Sciences, Universiti Putra Malaysia (UPM), Serdang, Selangor, Ministry of Higher Education, Malaysia; Clinical Research Centre, Institute of Clinical Research (NIH), Hospital Raja Permaisuri Bainun Ipoh, 30450, Perak, Ministry of Health, Malaysia; Clinical Research Centre, Institute of Clinical Research (NIH), Hospital Raja Permaisuri Bainun Ipoh, 30450, Perak, Ministry of Health, Malaysia; Clinical Research Centre, Institute of Clinical Research (NIH), Hospital Raja Permaisuri Bainun Ipoh, 30450, Perak, Ministry of Health, Malaysia; Department of Surgery (Urology), Sunway Medical Centre, Ipoh; Department of Surgery, Taylor’s University Subang Jaya, Selangor; Clinical Research Centre, Institute of Clinical Research (NIH), Hospital Raja Permaisuri Bainun Ipoh, 30450, Perak, Ministry of Health, Malaysia; ORL Department, Hospital Raja Permaisuri Bainun, Ipoh, 30450, Perak, Ministry of Health, Malaysia

**Keywords:** erectile dysfunction, prevalence, healthy men, IIEF-15, Malaysia

## Abstract

**Background:**

Erectile dysfunction (ED) is defined as the persistent inability to achieve or maintain an erection sufficient for satisfactory sexual performance, and in Malaysia, ED among the young is poorly understood.

**Aim:**

To identify the prevalence of ED in healthy Malaysian men aged 18 to 39 years and factors affecting the presence of ED.

**Methods:**

This cross-sectional study was conducted among healthy Malaysian men aged 18 to 39 years, using a standardized questionnaire. The link to the questionnaire was distributed via social media and communication applications. After consent, basic demography and ED was measured via the validated International Index of Erectile Function–15 (IIEF-15) questionnaire. Participants answered the questions in Malay or English. A sample size of 344 participants was needed for the study. Data was analysed using SPSS version 26.0 (IBM).

**Outcomes:**

To identify the prevalence of ED among young Malaysian men and its associated factors.

**Results:**

A total of 390 men completed the survey, and data from 361 were included in the final analysis. The mean age was 32.89 years (SD, 4.73), 66.5% were Malay, 49.6% were from the M40 group (middle 40% of wage earners in Malaysia), 52.4% were professionals in their fields, 31.3% were from Selangor, 67.6% lived in urban settings, 60.9% were married, 83.9% were heterosexually orientated, and the mean number of children among married men was 1.52 (SD, 1.24). The prevalence of ED was 32.7% (95% CI, 27.8%-37.9%) of the total participants, with 3.9% (95% CI, 2.2%-6.6%) experiencing severe ED. A multivariate regression analysis showed the following as being associated with ED: B40 status (below 40% of wage earners in Malaysia; adjusted odds ratio [AOR], 9.72; 95% CI, 2.43-38.94; *P* = .001), a homosexual/bisexual orientation (AOR, 11.66; 95% CI, 1.28-105.89; *P* = .03), and a decreased sexual desire (AOR, 9.02; 95% CI, 2.91-27.95; *P* < .001).

**Clinical Implications:**

Because ED was prevalent in younger Malaysians and linked to metabolic diseases, perhaps the screening of non-communicable diseases, including mental health, should also target younger Malaysians.

**Strengths and Limitations:**

This study was among the very few in Malaysia that examined ED in the young age group. However, factors such as smoking and psychosocial causes were not taken into account, as there may have been a potential reporting bias. We also included all sexual orientations into the study (i.e., IIEF-15 might have not been suitable for homosexuals — however, analysis removing the homosexual orientated group yielded similar results in the multivariate regression).

**Conclusion:**

The prevalence of ED in young healthy Malaysian men was about 1 in 3. Given the psychosocial factors involved, perhaps an insightful look toward mental health and socioeconomic affects to health should be considered for targeted screening programs.

## Introduction

### Background

Erectile dysfunction (ED) is a significant health issue affecting men globally, especially their quality of life, psychological well-being, and interpersonal relationships.^1^ Characterized by the persistent inability to achieve or maintain an erection sufficient for satisfactory sexual performance, ED is prevalent across various age groups, although its incidence increases with age.^2^ In primary care settings, ED often remains underreported and undertreated, despite its substantial prevalence and the availability of effective treatments.^3^^,^^4^ There is a factor known as early onset in ED such that patients of a younger age experience ED, which is poorly understood here in Malaysia.

In Malaysia, the prevalence and determinants of ED have been the subject of several studies, providing valuable insights into its epidemiology within this region. A study was conducted to determine the prevalence of ED in primary care settings among Malaysians, with the research highlighting the commonality of ED with significant predictors such as age, employment status, and comorbid conditions.^5^ However, a crucial limitation of the study was the inclusion of individuals with previously diagnosed comorbid conditions, which may have obscured the true prevalence of ED among men without known diagnoses.^5^

According to Ab Rahman et al, the prevalence of ED among Malaysian men in a primary care setting was significant, with aging being a predominant factor.^5^ As men age, the risk of developing ED increases — a trend consistent with global findings.^4^^,^^6^ Despite numerous insights, studies in Malaysia have not excluded individuals in their sample with any previously diagnosed comorbidities. This limitation highlighted the need for further research focusing on the prevalence of ED in Malaysians with undiagnosed comorbidities, especially non-communicable diseases (NCDs).

Employment status also plays a crucial role in the prevalence of ED. Unemployment and lower socioeconomic status are associated with higher rates of ED.^3^ ED is a prevalent and impactful condition in primary care settings in Malaysia, with significant links to age, employment status, and comorbidities. In Malaysia, B40, M40, and T20 refer to the local standardized socioeconomic groups, classifying income status as “below 40% of income earners,” “middle 40% of income earners,” and “top 20% of income earners.” A local Malaysian study that reported outcomes and subanalyzed samples close to the current study provides valuable insights, but underscores the need for further research focusing on undiagnosed cases to obtain a clearer understanding of ED’s true prevalence.^5^ This association may be attributed to the psychological stress and reduced access to health care resources experienced by unemployed individuals.^7^ Understanding the prevalence and associated factors of ED in a primary care context is essential for developing targeted interventions, and improving the overall management of this condition. Effective screening and diagnosis are crucial, particularly for undiagnosed cases, to ensure timely intervention and reduce the impact of ED on patients’ lives.^8^

By distinguishing between diagnosed and undiagnosed cases, health care providers can better identify at-risk populations and implement more effective screening and treatment strategies. Enhanced awareness, early identification, and comprehensive management of ED in primary care settings can significantly improve men’s health outcomes and overall well-being.

Few studies have examined ED in the younger age group. A study conducted among Brazilians in 2013 was the closest to the intention of this study. In that study, the authors found that 35.0% of men aged 18 to 40 years experienced ED.^9^ It also reported that ED was linked to lower educational qualifications, but not to sexual orientation, smoking, alcoholism, or any other comorbidities. For Malaysia, only a subset analysis was done for those aged <40 years. Most of the subset analyses were either inadequately powered or inadequately sampled to conclude the prevalence in the 18 to 39 age group.

Thus, it was first important to ask — what is the actual prevalence of ED among healthy 18- to 39-year-old Malaysians? ‘Healthy’ Malaysians in this research context referred to those without known comorbidities and included previously undiagnosed individuals. We included those with no known comorbidities because we believe that ED might be linked to comorbidities, and this is the group that is in danger of not being screened. From regular clinical observations, we find many young patients presenting with ED who are, unfortunately, being diagnosed with comorbidities after relevant clinical investigations. Individuals who were involved with sexual activity (i.e., penetrative sex) were important to be included in this research context to evaluate whether their ED was linked to sexual function, as this age range (18–39 years) represents a sexually active group.

### Objective

The main objective of this study was to identify the prevalence of ED among young and healthy Malaysian men aged 18 to 39 years. We also intended to evaluate the mean age of participants who developed ED and the factors associated with it.

## Methods

### Study design and setting

This cross-sectional study sampled healthy Malaysians aged 18 to 39 years. The questionnaire was distributed, collected, and collated online (via Google forms), making the method a form of convenient sampling.

### Participants

We sampled healthy Malaysian men aged 18 to 39 years. We selected this age group because it is deemed, from a local context, to be regularly free from comorbidities. Locally, this group happens to excluded from the government-led public health initiatives that provide complimentary yearly health screening packages for NCDs. Specifically, we included Malaysian men who were aged 18 to 39 years when answering the questionnaire, who were sexually active (had penetrative sex in the last 4 weeks), and had no known comorbidities.

We excluded those with the following conditions, as identified by previous research to be comorbidities or known factors that causes ED^5^:

- Hypertension
- Diabetes mellitus
- Ischemic heart diseases
- Dyslipidemia
- Parkinsonism
- Any form of cancers
- Neuropathy
- Genetic diseases (eg, Klinefelter’s)
- Penile trauma
- Peyronie’s disease
- Penile fractures
- Cavernous fibrosis

### Outcome

We intended to identify the prevalence of ED among young Malaysian men and the associated factors with ED.

### Data sources and measurement

We obtained data from participants via an online response mechanism. Data was collected from February to June 2025. Invitations to potential Malaysian participants were done via social media (WhatsApp©, Facebook©, Twitter©, and a many others). An online participant information sheet explained the study and its importance to potential respondents. Once participants agreed, they were taken to the page containing questions in the language of their choice (English or Bahasa Melayu (local language)). They were asked about their basic demographics, such as age, occupation, and existing comorbidities. They were then asked to answer the International Index of Erectile Function–15 (IIEF-15; the validated questionnaire in either Bahasa Melayu and English). They were allowed to take their time and answer the questionnaire according to their own pace. Participants were asked if they wanted to be contacted after the survey if they suffered from ED issues. They were then accordingly referred to the nearest health facility for subsequent screening and treatment. Data was collected and tabulated in Microsoft Excel before being imported into SPSS version 26.0 (IBM) for further analysis.

#### Bias

We acknowledge that the study has a risk of participation bias because it was sent to potential individuals via an online distribution mechanism.

#### Study size

Using the sample precision calculator in EpiCalc version 1.01 (2000), we calculated the sample size needed for this study. The final sample required for this study was 349. This was calculated by setting the precision at 5%, and the confidence interval at 95%, with an estimated prevalence of 34% — selected as it yielded the highest sample needed based on previous studies of younger men (18–40 years) with ED (0–34%).^2^^,^^9^

#### Questionnaire

The questionnaire used in this study is the IIEF-15, which has been validated in English and translated/ validated into Bahasa Melayu. It was made available for free by Dr Quek Kia Fatt from Monash University, Malaysia.^10^ The original questionnaire had a Cronbach α >0.90, with good internal and external reliability as well as good sensitivity and specificity.^11^ The Malay-translated version had a Cronbach α ≥0.74.^10^

#### Plan for data analysis and interpretation

All data that were categorical within the demographics were displayed as whole numbers and percentages. Percentages for the total data set are reported as column percentages and aggregated data according to outcomes (with and without ED) as row percentages. Continuous variables underwent normality testing: normal distribution was defined as having a skewness of ±1 and kurtosis of ±3. Normal data were displayed as mean and standard deviation (SD) while non-normally distributed data were reported as median and inter-quartile range (IQR). For the IIEF-15 questionnaire, the ED score ranged from 1 to 30, and the total score was classified as follows: severe ED (1–10), moderate (11–16), mild-to-moderate (17–21), mild (22–25), and no ED (26–30). We then re-categorized the data to a binary outcome– “no ED” (score 26–30) and “some form of ED” (score ≤25). A binary logistic regression was applied to identify variables of the demographic data that might be associated with the outcome of having ED. The univariate analysis was described as odds ratio (OR) and the multivariate analysis as adjusted OR (AOR). OR and AOR were reported with their corresponding 95% confidence intervals (CI). The univariate analysis included factors that were *P* ≤ .3 for the multivariate analysis. Statistical significance for the multivariate analysis was *P* < .05. The study was conducted according to the STROBE guidelines.

Data were analysed using SPSS version 26.0 (IBM).

#### Ethics

We registered the study with the National Medical Research Registry (NMRR) and obtained ethical approval from the Malaysian Research Ethics Committee (approval NMRR ID-24-03750-KMR [IIR]). Participation was voluntary, and an informed consent was taken from every participant before administration of the questionnaire (done online). No identifiers were collected, and all responses were dealt with in a highly confidential manner. For those who claimed to have ED and wanted help, we referred them to their nearest government health clinic for a thorough screening.

## Results

### Participants and descriptive data

A total of 390 men aged 18 to 39 years attempted the survey, of which 29 (7%) were dropped from the sample- for not agreeing to participate (n = 2, 0.5%) and either not having a partner or not having intercourse (n = 27, 6.9%).

For the final analysis- 361 participants were included (104.9% of the required sample size of 344).

The mean age of the participants was 32.89 years (SD, 4.73), and 66.5% were Malay. In addition, 51.8% had an undergraduate degree, 49.6% were in the M40 group, and 52.4% were professionals. Furthermore, 31.3% were from Selangor and 67.6% lived in urban settings, whereas 60.9% were married and 83.9% were of the heterosexual orientation. Finally, the median number of partners in the last month was 1.17 (IQR, 0), and the mean number of children among the married men was 1.52 (SD, 1.24). Full details can be seen in Table 1.

**Table 1:** Demography of participants consenting to the survey (N = 361).

**Variable**	**No. (%)**
Age, years, mean (SD)	32.89 (4.73)
Race	
Malay Chinese Indian Others	240 (66.5) 46 (12.7) 61 (16.9) 14 (3.9)
Highest qualification	
No formal education Primary school Secondary school Certificate Diploma Undergraduate Postgraduate No formal education/primary school/secondary school Certificate/diploma Undergraduate Postgraduate	1 (0.3) 1 (0.3) 15 (4.2) 5 (1.4) 49 (13.6) 187 (51.8) 103 (28.5) 17 (4.7) 54 (15.0) 187 (51.8) 103 (28.5)
Joint monthly income, RM/mo	
B40: ≤4849 M40: 4850-10 959 T20: ≥10 960	100 (27.7) 179 (49.6) 82 (22.7)
MASCO occupational classification	
Clerical support workers Armed forces occupations Elementary occupations Managers Plant and machine operators Service and sales workers Skilled agricultural workers Technicians Professionals Others	14 (3.9) 1 (0.3) 11 (3.0) 38 (10.5) 6 (1.7) 24 (6.6) 1 (0.3) 25 (6.9) 189 (52.4) 52 (14.4)
State	
Johor Kedah Kelantan Melaka Negeri Sembilan Pahang Perak Perlis Pulau Pinang Sabah Sarawak Selangor Terengganu WPKL WP Labuan WP Putrajaya	28 (7.8) 12 (3.3) 14 (3.9) 10 (2.8) 12 (3.3) 3 (0.8) 51 (14.1) 3 (0.8) 18 (5.0) 7 (1.9) 12 (3.3) 113 (31.3) 13 (3.6) 50 (13.9) 2 (0.6) 13 (3.6)
Area	
Urban Suburban Rural	244 (67.6) 105 (29.1) 12 (3.3)
Marital status	
Single Married Widower Divorced Single Married Widower/divorced	134 (37.1) 220 (60.9) 3 (0.8) 4 (1.1) 134 (37.1) 220 (60.9) 7 (1.9)
Sexual orientation	
Heterosexual	303 (83.9)
Homosexual	41 (11.4)
Bisexual Heterosexual Homo-/bisexual	17 (4.7) 303 (83.9) 58 (16.1)
No. of partners in the last month, median (IQR)	1.17 (0)
No. of children (married men only), mean (SD)	1.52 (1.24)

Abbreviation: MASCO, Malaysia Standard Classification of Occupations.

**Table 2:** Erectile dysfunction status, intercourse satisfaction, orgasmic function, sexual desire, and overall satisfaction of participants (N = 361).

**Variable**	**No. (%)**	**95% CI**
Erectile dysfunction status		
Normal With dysfunction Mild Mild to moderate Moderate Severe	243 (67.3) 118 (32.7) 38 (10.5) 42 (11.6) 24 (6.6) 14 (3.9)	27.8-37.9 62.2-72.1 7.6-14.2 8.6-15.5 4.4-9.8 2.2-6.6
Intercourse satisfaction		
Dissatisfied Satisfied	296 (82.0) 65 (18.0)	77.6-85.8 14.3-22.4
Orgasmic function		
Decreased No decrease	108 (29.9) 253 (70.1)	25.3-35.0 65.0-74.7
Sexual desire		
Decreased No decrease	238 (65.9) 123 (34.1)	60.7-70.7 29.3-39.3
Overall satisfaction		
Decreased No decrease	228 (63.2) 133 (36.8)	58.0-68.1 31.9-42.0

### ED status: outcome data

From the 361 respondents, 32.7% (n = 118; 95% CI, 27.8%-37.9%) had some form of ED (the prevalence of ED). Regarding the severity of the ED, 10.5% (95% CI, 7.6%-14.2%) had mild ED, 11.6% (95% CI, 8.6%-15.5%) mild to moderate, 6.6% (95% CI, 4.4%-9.8%) moderate, and 3.9% (95% CI, 2.2%-6.6%) severe.

From the total, 82.0% (95% CI, 77.6%-85.8%) had intercourse dissatisfaction, 29.9% (95% CI, 25.3%-35.0%) had a decrease in orgasmic function, 65.9% (95% CI, 60.7%-70.7%) had poor sexual desire, and 63.2% (95% CI, 58.0%-68.1%) had a decrease in overall satisfaction.

Full details are available in Table 2.

### Comparison of demographics between those with and without ED

A statistical test was performed to compare the findings between those with and without ED. From the comparison, we found that the following were significantly associated with the presence of ED: highest qualification (*P* = .002), joint monthly income (*P* < .001), occupational classification (*P* = .03), marital status (*P* = .01), sexual orientation (*P* = .01), intercourse satisfaction (*P* < .001), orgasmic function (*P* < .001), sexual desire (*P* = .002), and overall satisfaction (*P* < .001). However, as there was strong collinearity and interactions between ED and the domains of “intercourse satisfaction,” “orgasmic function” and “overall satisfaction,” they were excluded from the final multivariate analysis. The complete set of results can be seen in Table 3.

**Table 3:** Comparison between demographic variables of patients with and without erectile dysfunction (N = 361).^a^

	**Erectile dysfunction, No. (%)**		
**Variable**	**No (n = 243)**	**Yes (n = 118)**	***P* value**
Age, y, mean (SD)	32.96 (4.72)	32.74 (4.75)	.67
No. of partners in the last month, median (IQR)	1.00 (0)	1.00 (0)	.35
No. of children (married men), mean (SD)	1.56 (1.19)	1.41 (1.34)	.42
Race			
Malay Chinese Indian Others	163 (67.9) 30 (65.2) 40 (65.5) 10 (71.4)	77 (32.1) 16 (34.8) 21 (34.4) 4 (28.6)	.95^b^
Highest qualification			
No formal education Primary school Secondary school Certificate Diploma Undergraduate Postgraduate	0 0 7 (46.7) 4 (80.0) 25 (51.0) 127 (67.9) 80 (77.7)	1 (100) 1 (100) 8 (53.3) 1 (20.0) 24 (49.0) 60 (32.1) 23 (22.3)	**.002** ^b^
No formal education/primary school/secondary school Certificate/diploma Undergraduate Postgraduate	7 (41.2) 29 (53.7) 127 (67.9) 80 (77.7)	10 (58.8) 25 (46.3) 60 (32.1) 23 (22.3)	**.002**
Joint monthly income, RM/mo			
B40: ≤4849 M40: 4850-10 959 T20: ≥10 960	45 (45.0) 130 (72.6) 68 (82.8)	55 (55.0) 49 (27.4) 14 (17.1)	**<.001**
MASCO occupational classification			
Clerical support workers Armed forces occupations Elementary occupations Managers Plant and machine operators Others Service and sales workers Skilled agricultural workers Technicians Professionals	10 (71.4) 1 (100) 5 (45.50 24 (63.2) 1 (16.7) 34 (65.4) 14 (58.3) 0 14 (56.0) 140 (74.1)	4 (28.6) 0 6 (54.5) 14 (36.8) 5 (83.3) 14 (36.8) 10 (41.7) 1 (100) 11 (44.0) 49 (25.9)	**.03** ^b^
State			
Johor Kedah Kelantan Melaka Negeri Sembilan Pahang Perak Perlis Pulau Pinang Sabah Sarawak Selangor Terengganu WPKL WP Labuan WP Putrajaya	19 (67.9) 8 (66.7) 10 (71.4) 6 (60.0) 5 (41.7) 3 (100) 31 (60.8) 3 (100) 16 (88.9) 3 (42.9) 9 (75.0) 84 (74.3) 11 (84.6) 27 (54.0) 1 (50.0) 7 (53.8)	9 (32.1) 4 (33.3) 4 (28.6) 4 (40.0) 7 (58.3) 0 20 (39.2) 0 2 (11.1) 4 (57.1) 3 (25.0) 29 (25.7) 2 (15.4) 23 (46.0) 1 (50.0) 6 (46.2)	.07^b^
Area			
Urban Suburban Rural	170 (69.7) 68 (64.8) 5 (41.7)	74 (30.3) 37 (35.2) 7 (58.3)	.11
Marital status			
Single Married Widower Divorced	81 (60.4) 160 (72.7) 2 (66.7) 0	53 (39.6) 60 (27.3) 1 (33.3) 4 (100)	**.002** ^b^
Single Married Widower/divorced	81 (60.4) 160 (72.7) 2 (28.6)	53 (39.6) 60 (27.3) 5 (71.4)	**.01** ^b^
Sexual orientation			
Heterosexual Homosexual Bisexual	213 (70.3) 22 (53.7) 8 (47.1)	90 (29.7) 19 (46.3) 9 (5.9)	**.02** ^b^
Heterosexual Homo-/bisexual (non-heterosexual)	213 (70.3) 30 (51.7)	90 (29.7) 28 (48.3)	**.01**
Intercourse satisfaction			
Dissatisfaction Satisfaction	182 (61.5) 61 (93.8)	114 (38.5) 4 (6.2)	<.001^b^^,^^c^
Orgasmic function			
Decrease No decrease	31 (28.7) 212 (83.8)	77 (71.3) 41 (16.2)	<.001^b^^,^^c^
Sexual desire			
Decrease No decrease	147 (61.8) 96 (78.0)	91 (38.2) 27 (22.0)	**.002**
Overall satisfaction			
Decrease No decrease	119 (52.2) 124 (93.2)	109 (47.8) 9 (6.8)	<.001^c^

Abbreviation: MASCO, Malaysia Standard Classification of Occupations.

a Bold indicates *P* < .05.

b Exact test/Fisher exact test applied.

c Significant but not included in the final multivariate analysis due to strong collinearity with erectile dysfunction.

**Table 4:** Univariate and multivariate analysis to identify factors linked to erectile dysfunction (N = 361).^a^

**Variable**	**OR (95% CI)**	***P* value**	**AOR (95% CI)**	***P* value**
No. of children (only married men)	0.91 (0.72-1.15)	.42	0.79 (0.55-1.12)	.18
Highest qualification				
No formal education/primary school/secondary school Certificate/diploma Undergraduate Postgraduate	4.97 (1.70-14.51) 3.00 (1.48-6.09) 1.64 (0.94-2.87) 1 [Reference]	**.002** ^b^	1.10 (0.12-10.11) 2.35 (0.60-9.14) 2.70 (1.00-7.25) 1 [Reference]	.23
Joint monthly income, RM/mo				
B40: ≤4849 M40: 4850-10 959 T20: ≥10 960	5.94 (2.96-11.92) 1.83 (0.94-3.55) 1 [Reference]	**<.001**	9.72 (2.43-38.94) 1.38 (0.48-3.94) 1 [Reference]	**.001**
MASCO occupational classification				
Clerical support workers Armed forces occupations Elementary occupations Managers Others Plant and machine operators Service and sales workers Skilled agricultural workers Technicians Professionals	1.14 (0.34-3.81) <0.001 (<0.001, >1000) 3.43 (1.00-11.34) 1.67 (0.80-3.48) 1.51 (0.78-2.92) 14.29 (1.63-125.31) 2.04 (0.85-4.89) >1000 (<0.001, >1000) 2.25 (0.96-5.27) 1 [Reference]	**.03**	0.31 (0.03-2.80) <0.001 (<0.001, >1000) 6.96 (0.81-57.63) 0.99 (0.27-3.59) 2.28 (0.56-9.27) 3.15 (0.15-64.67) 0.79 (0.19-3.28) >1000 (<0.001, >1000) 0.95 (0.18-5.02) 1 [Reference]	.46
State				
Johor Kedah Kelantan Melaka Negeri Sembilan Pahang Perak Perlis Pulau Pinang Sabah Sarawak Selangor Terengganu WPKL WP Labuan WP Putrajaya	1 [Reference] 1.06 (0.25-4.45) 0.84 (0.21-3.44) 1.41 (0.32-6.27) 2.96 (0.73-11.93) <0.001 (<0.001, >1000) 1.36 (0.52-3.60) <0.001 (<0.001, >1000) 0.26 (0.05-1.40) 2.82 (0.52-15.32) 0.71 (0.15-3.25) 0.73 (0.30-1.79) 0.38 (0.07-2.11) 1.80 (0.68-4.74) 2.11 (0.12-37.72) 1.81(0.476.97)	**.07**	1 [Reference] 0.81 (0.07-8.82) 2.23 (0.21-23.75) 0.64 (0.02-25.26) 18.04 (0.69-474.56) <0.001 (<0.001, >1000) 1.54 (0.22-10.76) <0.001 (<0.001, >1000) 0.38 (0.02-7.03) 5.37 (0.37-77.12) 0.17 (0.01-5.30) 1.65 (0.27-10.27) 0.18 (0.01-4.96) 3.14 (0.38-25.71) 2.34 (0.05-107.52) 10.74 (0.85-135.34)	.49
Area				
Urban Suburban Rural	1 [Reference] 1.25 (0.77-2.03) 3.22 (0.99-10.46)	**.11**	1 [Reference] 1.72 (0.67-4.42) 1.17 (0.10-13.33)	.53
Marital status				
Single Married Widower/divorced	1 [Reference] 0.58 (0.36-0.90) 3.82 (0.72-20.42)	**.01**	1 [Reference] <0.001 (<0.001, >1000) 13.30 (1.00-177.44)	.05
Sexual orientation				
Heterosexual Homosexual/bisexual	1 [Reference] 2.21 (1.25-3.91)	**.01**	1 [Reference] 11.66 (1.28-105.89)	**.03**
Sexual desire				
Decrease No decrease	2.20 (1.33-3.63) 1 [Reference]	**.002**	9.02 (2.91-27.95) 1 [Reference]	**<.001**

Abbreviations: AOR, adjusted odds ratio; MASCO, Malaysia Standard Classification of Occupations; OR, odds ratio.

a Bold indicates *P* ≤ .3 for the univariate analysis and

^b^ *P* < .05 for multivariate analysis.

### Regression analysis

A binary logistic regression analysis was conducted to compare the demographic data with the outcome/dependent variable of “some form of ED.” The OR and AOR reflected the number of times that the variable was associated with “some form of ED” as compared with no ED. A 2-step univariate and multivariate analysis was conducted.

### Univariate analysis

Data were compared and the statistically significant variables (*P* ≤ .3) were as follows: highest qualification (*P* = .002), joint monthly income (*P* < .001), occupational classification per the Malaysia Standard Classification of Occupations (MASCO) (*P* = .03), the state in which participants resided (*P* = .07), area in which participants lived (*P* = .11), marital status (*P* = .01), sexual orientation (*P* = .01), and sexual desire (*P* = .002). Although the number of children among married men was not significant, it was entered into the final model as the researchers deemed it clinically important variable to the cause ED; specifically, the number of children could be less in those with ED (ie, infertility). The rest of the variables were excluded for not being statistically significant or for strong collinearity. These variables were entered into the multivariate analysis.

### Goodness-of-fit model

We conducted goodness-of-fit modeling for the final model of the variables included in the multivariate analysis. The Nagelkerke *R*^2^ yielded 0.51 (model fit ≥0.50); the Hosmer-Lemeshow test yielded *P* = .20 (good model >.05); and the correctly classified percentage was 81.5% (good model ≥80%). The final model was considered a good model.

### Multivariate analysis

The final model indicated that the likelihood of having ED was 9.72 times (95% CI, 2.43-38.94; *P* = .001) in the B40 group and 1.38 times (95% CI, 0.48-3.94; *P* = .01) in the M40 group as compared with the T20 group. Those who were non-heterosexually oriented (homosexual or bisexual) were 11.66 times (95% CI, 1.28-105.89; *P* = .03) more likely to have ED as compared with the heterosexual group. Those having decreased sexual desire were 9.02 times (95% CI, 2.91-27.95; *P* < .001) more likely to have ED as compared with those with normal sexual desire. Details are listed in Table 4. A few factors (eg, Perlis and those married) yielded an OR or AOR <0.001 (95% CI, <0.001 to >1000) perhaps due to their small numbers or samples (OR) or because the corrected AOR vs the reference was much smaller; these could not have been combined with other categories as they were simply independent.

### Removal of those who were non-heterosexual (homosexual/bisexual)

We understand that the IIEF-15 is discouraged to be used in those who are non-heterosexual. Thus, a separate analysis was conducted with the removal of the non-heterosexual participants. The results obtained were statistically significant in the same factors as with the inclusion of heterosexuals. There was a difference of up to 0.5 in the OR. The goodness-of-fit model, which correctly classified the percentage, was about the same as the previous model (81.7%). The Nagelkerke *R*^2^ yielded 0.44 (<0.50, poor fit), and the Hosmer-Lemeshow test was not significant (*P* = .93). It must be noted that the “number of partners” became significant in the univariate analysis (*P* = .15) but was not significant in the multivariate analysis.

## Discussion

### Key results

The prevalence of ED in 18- to 39-year-olds was 32.7% (95% CI, 27.8%-37.9%). Three notable factors were associated with ED. First, those having a joint monthly income of a B40 group were nearly 10 times more likely to have ED as compared with the T20 group. Second, those who were non-heterosexual (being homosexual or bisexual) were nearly 12 times more likely to have ED as compared with heterosexual men. Lastly, men who had decreased sexual desire were 9 times more likely to have ED as compared with those who had no reduction in sexual desire.

### Interpretation and generalization

Studies that support our findings are as follows. The prevalence of ED, as reported by a 1994 US study, was about 16% among those 18 to 39 years old.^12^ A similar study conducted in Brazil found that nearly 35% of healthy 18- to 40-year-olds experienced ED.^9^ This prevalence somewhat increased to about 20% as noted in a study conducted from 2001 to 2002.^13^ In Europe, a review revealed that the prevalence of ED among men <40 years old ranged from 1% to 25% depending on the sample and methods used.^14^ Another review reported that the prevalence of ED in the younger age group (<40 years) was as high as 30%, nearly similar to that found in this study.^15^

The more important question to ask is, what were the causes of ED in this age group, and why was it becoming common?

ED has been shown to be linked with many NCDs, such as cardiovascular health issues (cardiac failure and hypertension), metabolic conditions (dyslipidemia, diabetes), and even mental health/relationship issues.^9^^,^^14^^,^^16^^,^^17^

Previous literature reported that ED can be due to 2 main causes: psychogenic (largely attributed to mental health issues) or organic (hormonal, blood flow issues, structural abnormalities, neurologic disorders, surgery involving the pelvic floor, prostate causes), especially in the young.^15^^,^^17^

### Organic causes

From the various organic causes, ED can be related to metabolic conditions such as diabetes, high body mass index, dyslipidemia, smoking, sedentary lifestyle, and usage of recreational drugs and/or certain prescribed medications.^17^ What was even more worrying was that ED was strongly linked to cardiovascular diseases due to endothelial dysfunction as well as small vessel atherosclerosis.^17^ Considering the multiple associated factors, it is important that screening for these several comorbidities begin at an early stage so that prompt treatment and early control can be achieved. This is perhaps also a link with the actual hidden pandemic in NCDs.

From a 2010 study conducted in Brazil that screened 18- to 40-year olds, 35% of the 1947 individuals sampled had ED.^9^ From this total, 73.7% had mild ED and 26.3% had moderate to complete ED. From this Brazilian study, ED was not associated with level of education, race, employment status, and marital status, similar to our findings. However, the study found that sexual orientation was not linked to ED, which differed from the current study. An interesting bit of information was that lifestyle (smoking, alcoholism, obesity, sedentary lifestyle) was not linked to ED. This study concluded that ED caused a negative impact on a man’s self-esteem, interpersonal relationships, work performance, social activities, and sexual satisfaction. It also noted that <10% of those with ED had received any form of treatment. This Brazilian study showed that there might be a barrier for those with ED to obtain treatment.

However, a study that associated coronary velocity reserve with ED found a close relationship with the presence of diabetes, hypertension, dyslipidemia, and smoking, causing nearly 75% of all ED cases with coronary artery diseases. In a regression analysis, after adjusting for factors such as diabetes, hypertension, dyslipidemia, and others, ED was the only factor that was a statistically significant predictor of the reduced coronary flow velocity reserve. The study also reported that the IIEF-5 (the ED component of the IIEF-15) was associated with the total coronary artery plaque burden, which was confirmed via a coronary angiography. In a study discussing the causes and warnings of ED, the authors found ED to be an early marker for cardiovascular events, normally manifesting earlier than myocardial infarctions and stroke.^14^ As such, ED is sometimes used as a marker for cardiovascular diseases in younger men.^14^

From a study conducted in the northern United States, 16% of those aged 18 to 39 years had ED. Smoking and cardiovascular diseases were the factors that potentiated ED. It reported that ED was associated with having a chronic disease, especially diabetes mellitus (35%-75%). It was also interesting to note from a study conducted in Greece that young men with lower median intake of total flavonoids were more likely to have ED.^18^

### Psychogenic causes

From a study and narrative that examined psychosocial approaches to the treating of ED, the researchers concluded that there were no sufficiently validated models in high-quality experimental studies.^1^ This study found that partners of men with ED often report sexual problems, especially with intimacy and mental health, that might affect birth rates.^1^ In a review article, it was a cliché that ED was a self-limiting condition that, to many practitioners, did not warrant a proper clinical evaluation or specific therapy, which meant that many patients were offered clinical reassurance.^14^ A cause of psychogenic ED has been linked to depressive symptoms and was as high as 13%.^14^ Furthermore, depressive symptoms, anxiety performances, and some modern lifestyle habits are a cause of ED, especially in younger individuals.^13^^,^^14^^,^^19^ In another study conducted among young patients, it was reported that those that were depressed were likely to experience ED.^20^ The severity of ED also depended on the degree of depression.^20^

### Dietary intake

In a study conducted in Greece, men with ED had a lower median intake of total flavonoids, and the proper consumption of flavonoids per body weight lowered the risk of ED.^18^ The result of ED in the younger age group was due to poor dietary intake.^18^

### Burden of disease

The burden of disease for ED has inflated over the years in countries.^13^ One article reported that ED treatment expenditure by service site nearly doubled in 6 years and was expected to increase in the years to come.^13^ The cost of the burden of disease cannot be to better the erection function but to cure underlying causes such as uncontrolled diabetes, hypertension, dyslipidemia, and perhaps cardiac-related health problems. Thus, identifying the issue early, early intervention, and preventing debilitating diseases might be the way forward and might prove to be a more economically sustainable method. Although ED is perceived to be a self-limiting condition, proper assessment and a working diagnosis should be made to prevent worsening of the condition and complications. Other factors that could be linked to ED that might result in a high expenditure form of treatment are drug abuse and mental health issues.

Some of the causes of the ED can be due to mental health issues or can even cause issues with mental health. Having the burden of disease such as end-organ damage, sexual dysfunction, and other chronic diseases that can cause mental health diseases might give a silent rise to mental health conditions.^14^

### Strengths and weaknesses

#### Strengths

This study has notable strengths. First, it is among the few studies in Malaysia that has examined presumed healthy young age men and the presence of ED. Next, screening focused on young healthy Malaysians to see the potential burden of disease linked to ED. Finally, we required users to log into their Google accounts before answering to prevent duplicate responses by the same person.

#### Weaknesses

This study also has weaknesses that should be noted. First, some factors (eg, smoking) were not taken into account due to potential reporting bias. Next, data were collected online without professional supervision. Although we acknowledge that only people with internet connections would have answered (respondent bias and convenient sampling), the country’s internet access by 18- to 39-year-olds is well documented; they are the group that are internet savvy. Explanation of terminology and meaning of the questions were done within the questionnaire via definitions to reduce response errors by the respondents. This would have been the best way to get our target population to answer honestly and discreetly without feeling embarrassed (locally, ED is a taboo subject). In addition, we did not exclude sexual orientation in this study because we wanted to see if it was a factor. We acknowledge that the IIEF-15 was a poor predictor of ED in the homosexual group. This study also collected data online, which might have resulted in some selection bias. Moreover, there was no way to verify the age of the participants who participated. Last, the psychosocial factors of participants were not taken into account.

### Recommendations for future studies to consider

From the results in this study, we can say that 1 in 3 healthy Malaysian men aged 18 to 39 years has some form of ED. Perhaps, non-communicable conditions in Malaysians might be starting at early onset. This might require us to rethink our screening capacity and processes here in Malaysia. In Malaysia, screening for NCDs (PeKA B40 and PERKESO, both government-funded NCD screening programs) requires an eligible participant to be aged >40 years to qualify for the screening program. However, even with this screening, we are still see end-organ damage in Malaysians (eg, end-stage renal disease) continue to rise in numbers. As such, screening early might assist the health care system to detect patients in their pre-NCD stage to start managing the disease or even preventing it altogether. As we know from different studies, ED is a predictor for cardiovascular diseases, including strokes, myocardial infarctions, and other end-organ damages. Screening and controlling NCDs such as diabetes, hypertension, and renal function disorders from an early stage will assist in preventing end-artery complications.

## Conclusion

The prevalence of ED in young healthy Malaysians was 1 in 3. Factors that were associated with ED included being in the B40 group, having a non-heterosexual orientation, and having reduced sexual desire. As these are psychosocial factors, perhaps an insightful look toward mental health and the socioeconomic effects to health should be revisited. It might also be a consideration for better screening programs, including opportunistic screening, in mental health and other NCDs.
